# Ocular Surface Temperature Profile of Eyes with Retinal Vein Occlusion

**DOI:** 10.3390/jcm12237479

**Published:** 2023-12-03

**Authors:** Shany Shperling, Tommy Mordo, Gabriel Katz, Amir Alhalel, Alon Skaat, Gal Yaakov Cohen, Ofira Zloto, Ari Leshno

**Affiliations:** 1 Goldschleger Eye Institute, Sheba Medical Center, Tel Hashomer, Ramat Gan 5262000, Israelgabrielkatz.dr@gmail.com (G.K.); amir.alhalel@sheba.health.gov.il (A.A.); alon.skaat@sheba.health.gov.il (A.S.); ozloto@gmail.com (O.Z.); 2 Adelson School of Medicine, Ariel University, Ariel 40700, Israel; 3 Sackler Faculty of Medicine, Tel Aviv University, Tel Aviv 69978, Israel; 4 The Sheba Talpiot Medical Leadership Program, Sheba Medical Center, Tel Hashomer, Ramat Gan 52621, Israel

**Keywords:** ocular surface temperature, retinal vein occlusion, thermography

## Abstract

Retinal vein occlusion (RVO) results in ischemia followed by an inflammatory response. Both processes affect tissue temperature in opposite directions. Here, we evaluate the effect of RVO on the ocular surface temperature (OST) profile. Subjects with RVO were prospectively recruited. Healthy subjects without any ocular disease served as controls. The OST was determined using the Therm-App thermal imaging camera, and image processing software was employed to compute the mean temperature values of the medial canthus, lateral canthus, and cornea. We obtained thermographic images from 30 RVO subjects (30 eyes) and 148 controls (148 eyes). A univariate analysis found that eyes with RVO had significantly elevated OSTs compared to the controls (mean difference of 0.6 ± 0.3 Celsius, *p* < 0.05). However, this distinction between the groups lost statistical significance upon adjusting for possible confounders, including patient and environmental factors. These findings were confirmed with a post hoc case–control matched comparison. In conclusion, RVO does not seem to affect the OST. This might be due to the balance between inflammatory thermogenesis and heat constriction from ischemia in RVO. It is also possible that, in our cohort, the RVO pathophysiological processes involved were localized and did not extend to the anterior segment. Patient and environmental factors must be considered when interpreting the OST.

## 1. Introduction

Retinal vein occlusion (RVO) is the second most common retinal vascular disorder, and may cause hemorrhage, edema, and, in extreme cases, blindness [1,2]. Branch retinal vein occlusion (BRVO) is the most prevalent form of this disease, affecting 0.4% of the world’s population, whereas central retinal vein occlusion (CRVO) is less common, with a prevalence of only 0.08% [3]. The pathophysiology of RVO originates from an acute vascular event that leads to an inflammatory reaction [4]. Although the disease is identified in the posterior segment, the association with systemic and ocular risk factors suggests that the rest of the globe might also be affected [5,6]. This hypothesis is supported by previous work showing that other retinal disorders (e.g., diabetic retinopathy and age-related macular degeneration) can have an effect on anterior segment properties, expressed by changes in the ocular surface temperature (OST) [7]. Both inflammation and ischemia affect tissue temperature in opposite directions. Given the powerful combination of both processes in RVO, the net effect on temperature highlights that component that is more significant.

Among the many uses of thermal imaging in various fields, this modality can also be used in medicine to identify vascular, inflammatory, rheumatological, and neoplastic pathologies [8,9,10]. In other medical disciplines, the organ of interest may be covered by surrounding tissues; however, ophthalmology has the advantage that this technology can be directly applied to gauge the ocular surface temperature. Although the OST can be affected by various non-ocular factors (e.g., body temperature and environmental features), previous work has shown that when these factors are taken into account, OST can be a useful biomarker of ocular hemodynamics in various ocular conditions and provides insights into their pathophysiology [7,11,12,13,14].

While there is some evidence to suggest that OST might also be affected by the presence of RVO, the exact nature of OST in the setting of RVO has not been fully elucidated [15]. This study’s main purpose is to unveil the OST profile of eyes affected by RVO. Our results suggest that the effect of RVO on OST might be negligible.

## 2. Materials and Methods

### 2.1. Study Design and Study Population

This was a cross-sectional study. The study followed the tenets of the Declaration of Helsinki and was approved by the institutional Review Board at Sheba Medical Center. Informed consent was obtained from each study participant after an explanation of the purpose of the study and description of its procedures. 

Patients diagnosed with RVO in one eye who were treated and followed up at the Sheba Medical Center during the period of January–June 2022 were prospectively recruited. Subjects were suitable for enrollment if they had unilateral RVO. Excluded patients had concomitant retinal pathologies discovered during their ophthalmic medical histories or underwent eye surgery six months prior to the examination. Patients with unaffected eyes served as the control group. In addition, a healthy control group was included, being composed of 148 individuals who had a normal eye exam during their annual screening examination [11].

### 2.2. Thermographic Image Capturing

**Figure A1:**
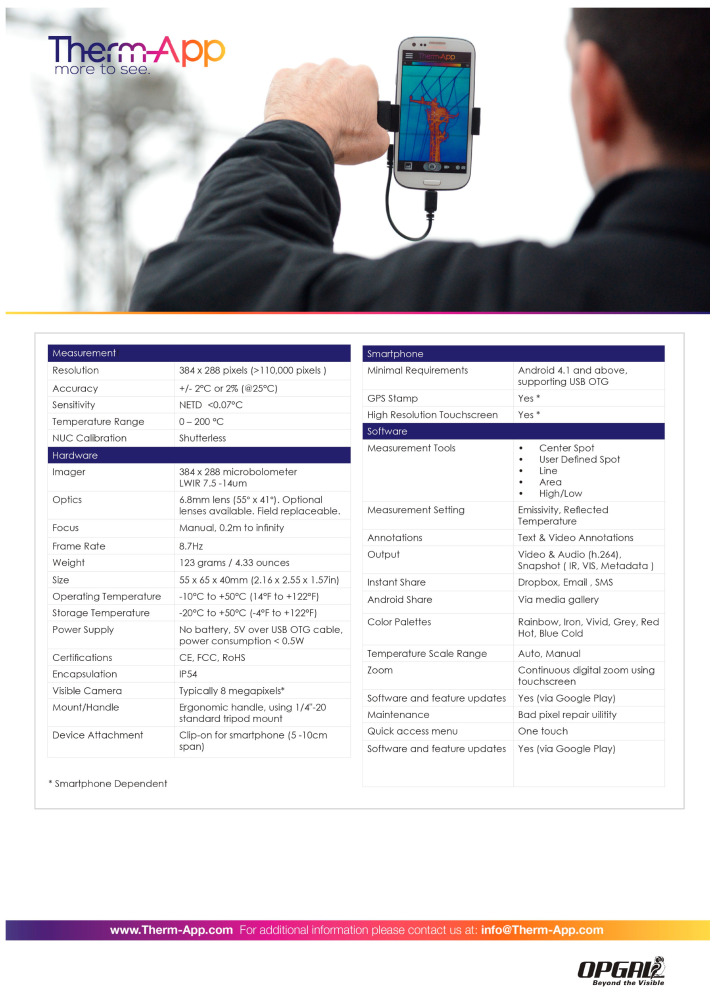
Detailed specifications of the Therm−App TH thermographic camera.

Thermographic imaging was performed in a controlled environmental condition, which included an artificially lit, windowless room with a central air-conditioning system with a constant setting, maintaining the room temperature at 23 degrees Celsius. Prior to the image capturing, measurements of the room temperature, humidity, and oral body temperature were obtained, and OST measurements were recorded with a Therm-App camera (TH^®^, Opgal Optronic Industries Ltd, Karmiel, Israel) with a 9 mm lens (384 × 284-pixel resolution). The full specifications are available in Figure A1. Thermographic images were taken after an adequate acclimatization time (20 min) to the room’s parameters. The participants were positioned in a standard slit-lamp head rest during the image capturing, and the Therm-App^®^ camera was positioned to directly face the orbit using a custom-made stabilizing arm connected to a head rest. After ensuring the proper positioning of the head and camera, the participants were instructed to keep their eyes closed for 10 s prior to the image capture, and upon eyelid opening, to fixate on the center of the camera lens. The picture was taken immediately after they opened their eyes in order to minimize any tear film evaporation effect. Based on past experiences, despite our best attempts to fully control the room conditions, some variability was expected. Given that environmental factors can significantly affect OST measurements (e.g., a 1 °C increase in room temperature leads to a 0.21 °C increase in the corneal OST), factors such as room temperature and humidity were recorded prior to any thermographic image capturing for adjustment [16]. Thermal imaging was performed for both eyes of patients in the RVO group and in the right eyes of all subjects in the control group. In order to avoid any possible side effects of the treatment, all images were taken at least 30 days following the most recent intravitreal injection.

### 2.3. OST Measurements

**Figure 1:**
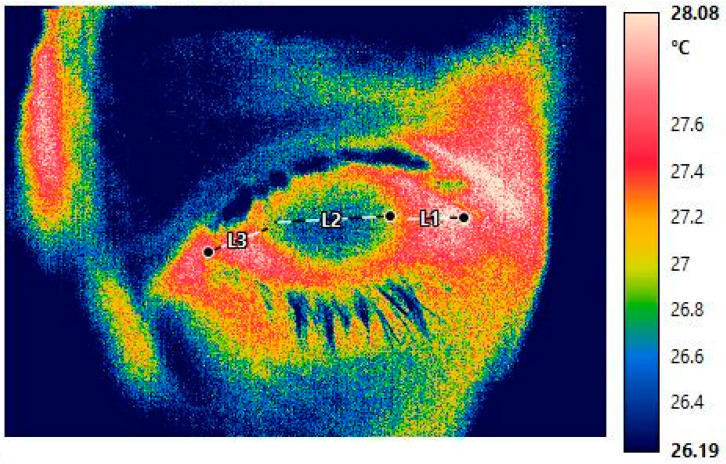
Example of measurements of ocular surface temperature. The dashed line follows the lateral, central (cornea), and medial regions (L1, L2, and L3, respectively).

The OST measurements were retrieved from the thermographic images by means of IRT cornista^®^ 4.0 software (GRAYESS Inc. 1903 60th place, Bradenton, FL, USA). For each image, three lines were drawn to obtain the average temperature (Celsius) at three ocular regions: (1) between the nasal limbus and the medial canthus (medial region), (2) between the horizontal edges of the temporal and nasal limbus (central region), and (3) between the lateral canthus and temporal limbus (lateral region) (Figure 1). This methodology was based on previous publications that found a measurement of a single area should not be considered as a reference for the average OST [17,18]. Image quality was evaluated and determined “adequate” based on our ability to identify major anatomical landmarks such as the corneal limbus and plica semilunaris.

### 2.4. Statistical Analysis

Continuous variables are shown as mean ± standard error (SE) and categorical variables as a frequency and percentage. Comparison of demographic and clinical data between groups was performed with a t-test for independent continuous variables and a two-sided Fisher’s exact test for categorical variables. A t-test for dependent continuous variables was used to examine the OST differences between affected and unaffected eyes in different regions. Multivariate regression models were employed in order to rule out a possible confounder effect of demographic, clinical, and environmental factors. Potential confounders which might affect OST were first identified by univariate analysis: for continuous variables, Pearson’s correlations coefficients with OST measurements were calculated; and for categorical variables, we used two independent-sample t-tests to compare OST measurements based on the presence or lack of each feature. Variables that were found to have a statistically significant association with OST in the univariate analysis were included as co-factors in the multivariate regression model. The statistical analysis was calculated using python3 with the following packages: scipy.stats and statsmodels. Statistical significance was set as *p* < 0.05.

## 3. Results

A total of 36 RVO patients were recruited. All the patients were diagnosed and treated with intravitreal anti-VEGF injections for RVO in only one eye (left = 12; right = 18). Six patients were excluded due to the presence of glaucoma (3), hypertensive retinopathy (1), history of posterior vitrectomy (1), and amblyopia (1) in at least one eye. Of the 30 remaining participants, 16 were diagnosed with CRVO and 14 with BRVO. No significant differences were observed between the CRVO and BRVO groups in demographics and clinical characteristics, or the OST profiles in both their affected and unaffected eyes (Table A1). Therefore, the two groups were combined and defined as the RVO group.

### 3.1. RVO-Affected vs. Unaffected Eyes

No significant differences in OST were observed between the RVO eye compared to the fellow control eyes (*p* > 0.75) in any of the three measured regions (Table 1). Furthermore, subgroup analysis of the BRVO and CRVO eyes separately did not reveal any significant differences in OST compared to the fellow eye (Figure A1).

### 3.2. Study Patients vs. Healthy Controls 

Several statistically significant differences were identified between the patients in the healthy control group and those in the RVO group, including demographics and the prevalence of systemic diseases. Most notably, RVO patients were older (age mean ± SD 65.9 ± 11.4 vs. 51.9 ± 10.2, *p* < 0.001) and had a higher prevalence of systemic vascular diseases, including hypertension (66.7% vs. 15.5%, *p* < 0.001), and cerebral vascular accident (CVA)/transient ischemic attack (TIA) (16.7% vs. <1%, *p* < 0.001). Details of the comparison are provided in Table 2.

The OST in RVO eyes was, on average, higher compared to that in eyes in the healthy control group. The mean difference was the greatest in the central region (0.7 ± 0.3°, *p* = 0.006) followed by the medial (0.6 ± 0.3°, *p* = 0.03) and lateral (0.5 ± 0.3°, *p* = 0.05) regions (Table 2). A similar pattern of difference in OST was also observed between the unaffected eyes of the RVO patients and eyes of healthy controls, with a mean difference of 0.7 ± 0.3° (*p* < 0.01) in the central and medial regions, followed by 0.4 ± 0.3 (*p* < 0.2) in the lateral region. 

The univariate analysis identified several variables that significantly correlated with OST measurements (Table A2 and Table A3). Most notably, sex, environmental factors (e.g., room temperature), and systemic illness (e.g., anemia and history of CVA or TIA) were found to have a significant association with OST in at least one region. To rule out the possibility that these factors led to a confounder effect, a multivariate logistic regression model was applied. Variables found to be significantly associated with OST in the univariate analysis were included in the model in a stepwise manner. The model revealed that there was no significant association between the presence of RVO and OST after adjustment for room temperature, sex, anemia, and history of CVA or TIA (Table 3 and Table A4). 

### 3.3. Case–Control

**Figure 2:**
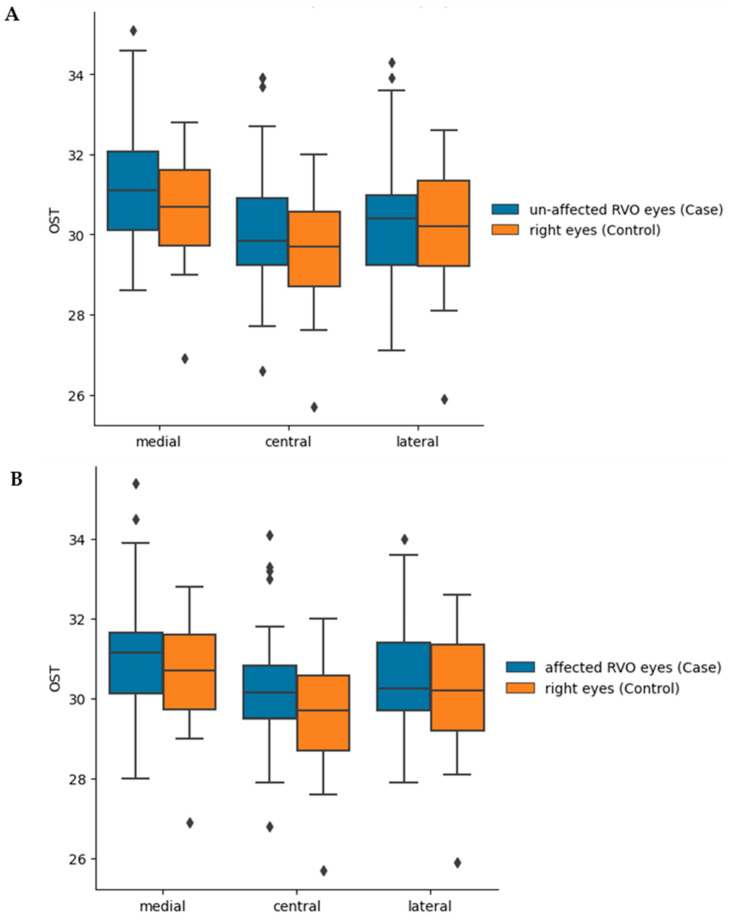
(**A**) Case–control analysis—OST of affected eyes of RVO patients vs. eyes of healthy control patients. (**B**) OST of unaffected eyes of RVO patients vs. eyes of healthy control patients. The dots represent the anomalies within the samples of OST; the bars represent maximum and minimum OST values (with exclusion of the anomalies). The upper part of the box represents the 75th percentile, the lower part represents the 25th percentile, and the line between them is the median.

In order to verify the results of the multivariate regression analysis and minimize the demographic variance, we performed a post hoc matched case–control comparison: 26 patients among the RVO group were matched to healthy controls by sex, age (within a 4-year range), and systemic medical history (e.g., presence of hypertension or history of prior CVA). Due to the relatively small sample size, non-parametric tests were used, and the comparison was evaluated using the Mann–Whitney *U* test. Compared to the healthy control group, the OST was higher in both the affected and non-affected eyes of the RVO group (Figure 2), but the difference did not reach statistical significance (Table A5 and Table A6).

## 4. Discussion

The primary objective of this study was to evaluate the OST profile of RVO eyes using ocular thermography. By analyzing the OST of affected unilateral RVO eyes and comparing them to the fellow unaffected eyes, as well as by comparing RVO patients to a sample of healthy controls, we concluded that RVO has no significant effect on OST.

In contrast to our findings, Sodi et al. [18] observed a significant difference in OST between healthy eyes and eyes afflicted with RVO. While their findings seem to coincide with other studies related to the pathophysiology of RVO, the fact that we were not able to replicate their results warrants clarification. This dissimilarity can be attributed to several methodological variances between the two studies. First, the different outcome seems to result from the distinct statistical approaches employed in each study. Specifically, the absence of a multivariate analysis in the study by Sodi et al. stands out. Both studies exhibited a significant difference in OST between the subject groups. However, the multivariate analysis employed in our study revealed that these variances were only secondary to a confounder effect. There were no statistically significance findings between the two groups following adjustment for environmental factors and patient characteristics. 

This type of adjustment has been implemented in several previous studies demonstrating how logistic regression analysis can aid in assessing the extent to which a particular condition impacts OST [7,12,19,20,21,22]. Furthermore, in Sodi et al., statistical significance was reached at only one of the five points measured. Another point worth mentioning is that both our univariate and multivariate analyses indicated higher OST in the RVO group than the controls, whereas Sodi et al. [18] found the RVO eyes to be colder. This implies the presence of additional potential technical distinctions between the two studies. For example, the percentage of male subjects was much higher in our RVO cohort (73% vs. 42%). In addition, they used a relatively older thermal camera (Agema Thermovision^®^ 800 LWB, AGEMA infrared Systems 1991 AB, Donderyd, Sweden), while different software was employed to capture images and extract OST measurements. Although we did not find that the presence of RVO has a significant effect on OST, prior research has demonstrated that OST can be affected by other posterior segment conditions, such as AMD, DME, and DR [7]. The lack of change in the OST in RVO eyes might highlight the underlying pathophysiology and potential differences from other retinal conditions. While the pathophysiology of both DR and AMD involves both ischemia and inflammation, changes in OST may be the result of the more dominant factor in each condition. Our observation that OST is not significantly affected by RVO suggests a balance between the ischemic and inflammatory processes in this disorder. In addition, this difference might be related to sequence of events in each condition. While both RVO and DR are related to impairment of the retinal vasculature, which can lead to cystic macular edema, the former is attributed to acute thrombotic incidents and localized retinal damage, whereas the latter stems from chronic systemic vascular changes [2,23,24]. Consequently, RVO may not necessarily impose a significant effect on other ocular tissues and thus may not influence OST to the same degree. 

Our study encountered a few limitations. First, compared to previous similar studies, the sample size of the RVO study group was relatively small. Therefore, the study was underpowered and thus unable to detect minor effects that RVO might have had on OST. However, the consistency of our results in both the multivariate analysis and the post hoc matched case–control analysis supports the conclusion that such an effect would be negligible. The small sample size of RVO patients also made it challenging to deduce differences between the BRVO and CRVO groups. A larger sample is needed to validate our findings as well as assess the extent to which they can be generalized to each RVO subtype. Secondly, establishing a controlled environment to address confounding variables proved to be difficult. For example, disparities in the seasons during which the measurements were obtained between RVO patients and healthy controls could have influenced variables such as room temperature or humidity. In addition, the patients in our cohort were all treated with anti-VEGF injections during thermography image acquisition. To the best of our knowledge, the effect of this treatment on OST has yet to be investigated, and the OST measurements of untreated RVO eyes might be different than those found herein. Additional research is needed to further assess the correlation between OST and other hemodynamic parameters, such as arterial pulse or amplitude, blood coagulation markers, ocular autonomic function, retinal features on optical coherence tomography, and retinal blood perfusion parameters in healthy eyes and eyes with various ocular co-morbidities. Our analysis was based on simple metrics that were used to estimate the OST in three designated regions. While this approach has been commonly used in ocular thermal imaging studies, a different approach to evaluating the thermographic profile may have yielded better results in identifying differences in OST. In addition, the use of artificial intelligence image analysis techniques might also provide new insights into the potential use of thermal imaging and possibly improve the value of this technology for the detection and management of patients.

## 5. Conclusions

This study did not find a significant effect of RVO on OST. Despite the possible limitation discussed above, our results indicate that the effect of RVO on OST is not clinically significant. This suggests that vascular changes are either counterbalanced by the inflammatory response in RVO or that the changes are mainly confined to the posterior segment. In any case, this study suggests that current thermal imaging techniques would not be useful in the management of this condition. Our analysis also highlights the importance of adjustment for the possible influence of environmental factors when interpreting OST measurements. Further studies should be conducted to investigate naïve patients as a study group, with efforts to reduce the influence of confounding variables while increasing the sample size.

## Figures and Tables

**Table 1:** Descriptive statistics of OST measurements of affected eyes, unaffected eyes, and control eyes.

	1. RVO	2. Unaffected by RVO	3. Healthy Controls	*p*-Value (1 vs. 2)	*p*-Value (2 vs. 3)	*p*-Value (1 vs. 3)
Medial	31.3 ± 1.8 (27.6–35.4)	31.4 ± 1.8 (28.6–35.1)	30.7 ± 1.1 (26.9–33.0)	*p* = 0.83	*p* = 0.01	*p* = 0.03
Central	30.4 ± 1.8 (26.8–34.1)	30.4 ± 1.8 (26.6–33.9)	29.7 ± 1.2 (25.7–32.0)	*p* = 0.87	*p* = 0.01	*p* = 0.006
Lateral	30.7 ± 1.8 (27.7–34.0)	30.6 ± 1.8 (27.1–34.4)	30.2 ± 1.1 (25.9–32.6)	*p* = 0.75	*p* = 0.16	*p* = 0.05

Descriptive statistics of OST measurements in all ocular regions of affected eyes of the study group vs. unaffected eyes of study group vs. right eyes of control group. *p*-value (independent t-test) of OST in all ocular regions of unaffected and affected eyes vs. control group right eyes. Dependent t-test was calculated, comparing OST in corneal regions of unaffected and affected eyes.

**Table 2:** Descriptive demographics of control and experiment participants.

Demographic Characteristics	Variable	*n* = 148 (Control)	*n* = 30 (RVO)	*p*-Value
	Age (year)	51.9 ± 10.2	65.9 ± 11.4	<0.001
	Male sex, *n*	90 (60%)	22 (74%)	0.22
Medical history, *n*				
	HTN	23 (15.5%)	20 (66.7%)	<0.001
	DM	11 (7.4%)	8 (26.7%)	0.006
	Anemia	9 (6.1%)	5 (16.7%)	0.07
	Dyslipidemia	121 (81.8%)	14 (48.3%)	0.68
	OSA	5 (3.4%)	1 (3.4%)	1.0
	IHD	8 (5.4%)	2 (6.7%)	0.68
	CVA/TIA	1 (<1%)	5 (16.7%)	<0.001
	DVT	X	1 (3.4%)	X
Physical examination				
	Systolic BP (mmHg)	124.3 ± 20.0	148.7 ± 17.8	<0.001
	Diastolic BP (mmHg)	75.6 ± 10.8	85.5 ± 15.7	<0.001
	HR (bpm)	70.3 ± 14.3	76.4 ± 12.4	0.05
	Body temperature (°C)	36.6 ± 0.2	36.5 ± 0.9	0.5
	Room temperature (°C)	21.6 ± 1.6	23.1 ± 1.0	<0.001
	Humidity (%)	55.8 ± 11.1	50.6 ± 12.3	0.03

Descriptive demographics of control and experiment participants. *p*-value was calculated (independent t-test or quantitative variables and Fisher test for categoric variables). Abbreviations: HTN, hypertension; DM, diabetes mellitus; OSA, obstructive sleep apnea; IHD, ischemic heart disease; CVA, cerebral vascular accident; TIA, transient ischemic attack; DVT, deep vein thrombosis; HR, heart rate; BP, blood pressure.

**Table 3:** Multivariate logistic regression analysis for OST and confounding variables.

	Dependent Variable: OST in the Medial Canthal Region			
Variable	OR	95% CI		*p*-Value
		Lower	Upper	
Sex (1 = male; 0 = female)	1.7	0.19	4.17	0.25
Room Temperature (°C)	4.5	1.53	2.69	<0.001
Anemia (1 = yes; 0 = no)	2.1	0.6	7.08	0.25
CVA/TIA (1 = yes; 0 = no)	12.9	1.42	118.33	0.02
OST(°C)	0.9	0.66	1.23	0.51
	Dependent variable: OST in the central corneal region			
Variable	OR	95% CI		*p*-Value
		Lower	Upper	
Sex (1 = male; 0 = female)	1.72	0.69	4.28	0.24
Room Temperature (°C)	2.03	1.53	2.69	<0.001
Anemia (1 = yes; 0 = no)	2.09	0.61	7.18	0.24
CVA/TIA (1 = yes; 0 = no)	13.02	1.43	118.89	0.02
OST(°C)	0.91	0.67	1.23	0.53
	Dependent variable: OST in the lateral canthal region			
Variable	OR	95% CI		*p*-Value
		Lower	Upper	
Sex (1 = male; 0 = female)	1.59	0.67	3.78	0.29
Room Temperature (°C)	2.17	1.64	2.88	<0.001
Anemia (1 = yes; 0 = no)	2.44	0.75	7.95	0.14
OST(°C)	0.74	0.54	1.0	0.06

Multivariate logistic regression analysis for OST and confounding variables. The comparison was made between the right eye of the control group and the affected and unaffected eyes of the experiment. Multivariate logistic regression with adjustment for sex, room temperature, anemia and for medial and central—also CVA. Abbreviations: OST, ocular surface temperature; CI, confidence interval; OR, odds ratio.

## Data Availability

Data are available upon reasonable request.

## Appendix A

**Table A1:** Descriptive demographic of BRVO and CRVO patients.

Demographic Characteristics	Variable	*n* = 14 (BRVO)	*n* = 16 (CRVO)	*p*-Value
	Age (year)	66.1 ± 10.0	65.8 ± 12.7	0.94
	Male sex, *n*	11 (78%)	11 (68%)	0.69
Medical history, *n*				
	HTN	9 (64%)	11 (69%)	1.0
	DM	3 (21%)	5 (31%)	0.69
	Anemia	1 (7%)	4 (25%)	0.34
	Dyslipidemia	9 (64.0%)	5 (33%)	0.14
	OSA	0 (0%)	1 (6%)	1.0
	IHD	0 (0%)	2 (12%)	0.48
	CVA/TIA	3 (<1%)	2 (16.7%)	0.64
	DVT	1 (7%)	0 (0%)	0.47
	IOP (sick eye)	15.2 ± 3.1	17.0 ± 3.1	0.95
Physical examination				
	Systolic BP (mmHg)	153.4 ± 18.5	143.7 ± 16.3	0.95
	Diastolic BP (mmHg)	89.7 ± 11.6	81.7 ± 18.3	0.95
	HR (bpm)	79.2 ± 13.1	73.7 ± 11.7	0.95
	Body temperature (°C)	36.3 ± 1.1	36.7 ± 0.6	0.95
	Room temperature (°C)	23.3 ± 1.3	22.8 ± 0.8	0.95
	Humidity (%)	49.4 ± 12.7	51.7 ± 12.3	0.95
	Medial OST of affected eyes	31.4 ± 1.3 (29.8–34.8)	31.2 ± 2.2 (27.6–35.4)	0.69
OST	Central OST of affected eyes	31.4 ± 1.3 (29.8–34.8)	30.3 ± 2.1 (26.8–34.1)	0.67
	Lateral OST of affected eyes	31.0 ± 1.5 (28.6–33.8)	30.5 ± 2.0 (27.7–34.0)	0.47

Descriptive demographic of BRVO and CRVO patients. *p*-value was calculated (independent t-test or quantitative variables and Fisher test for categoric variables). Abbreviations: HTN, hypertension; DM, diabetes mellitus; OSA, obstructive sleep apnea; IHD, ischemic heart disease; CVA, cerebral vascular accident; TIA, transient ischemic attack; DVT, deep vein thrombosis; HR, heart rate, IOP, intra-ocular pressure; DVT, deep vein thrombosis; BP, blood pressure; OST, ocular surface temperature of affected eyes.

**Table A2:** Affected, unaffected, and healthy controls eyes vs. patient and environmental characteristics correlations with OST.

	Medial Canthus		Central Cornea		Lateral Canthus	
Patient Characteristics:						
Age (years)	*r* = −0.08	*p* = 0.29	*r* = −0.03	*p* = 0.67	*r* = −0.08	*p* = 0.29
Body temperature (°C)	*r* = −0.08	*p* = 0.25	*r* = −0.09	*p* = 0.22	*r* = −0.07	*p* = 0.34
Systolic BP (mmHg)	*r* = −0.01	*p* = 0.94	*r* = 0.05	*p* = 0. 5	*r* = −0.03	*p* = 0.76
Diastolic BP (mmHg)	*r* = 0.07	*p* = 0.33	*r* = 0.11	*p* = 0.13	*r* = 0.05	*p* = 0.52
Maximal HR (bpm)	*r* = 0.06	*p* = 0.42	*r* = 0.06	*p* = 0.38	*r* = 0.04	*p* = 0.58
Environmental Characteristics						
Room temperature (°C)	*r* = 0.42	*p* < 0.01	*r* = 0.43	*p* < 0.01	*r* = 0.4	*p* < 0.01
Humidity (%)	*r* = 0.01	*p* = 0.86	*r* = 0.01	*p* = 0.87	*r* = 0.04	*p* = 0.61

Affected, unaffected, and healthy control eyes patient and environmental characteristics correlations with OST. Abbreviations: BP—blood pressure; HR—heart rate.

**Table A3:** OST comparison for categoric variables of affected, unaffected, and healthy control right eyes.

Variable (*n*)	Medial Canthus		Central Cornea		Lateral Canthus	
Sex		*p* = 0.02		*p* < 0.01		*p* = 0.02
Males (133)	31.1 ± 1.3		30.2 ± 1.4		30.5 ± 1.3	
Females (74)	30.6 ± 1.4		29.5 ± 1.4		30.1 ± 1.3	
HTN		*p* = 0.33		*p* = 0.1		*p* = 0.4
No (140)	30.9 ± 1.3		29.8 ± 1.3		30.3 ± 1.2	
Yes (63)	31.1 ± 1.6		30.2 ± 1.7		30.5 ± 1.6	
DM		*p* = 0.57		*p* = 0.19		*p* = 0.82
No (176)	30.9 ± 1.4		29.9 ± 1.5		30.4 ± 1.4	
Yes (27)	31.0 ± 1.1		30.3 ± 1.2		30.4 ± 1.2	
Dyslipidemia		*p* = 0.74		*p* = 0.76		*p* = 0.49
No (56)	30.8 ± 1.4		29.9 ± 1.5		30.25 ± 1.4	
Yes (149)	30.9 ± 1.4		29.9 ± 1.4		30.4 ± 1.3	
Anemia		*p* < 0.01		*p* < 0.01		*p* = 0.03
No (183)	30.8 ± 1.3		29.8 ± 1.3		30.3 ± 1.3	
Yes (19)	31.7 ± 2.1		30.8 ± 2.0		31.0 ± 2.0	
CVA/TIA						
No (192)	30.9 ± 1.3	*p* = 0.02	29.9 ± 1.4	*p* = 0.03	30.3 ± 1.3	*p* = 0.07
Yes (11)	31.8 ± 2.2		30.8 ± 2.2		31.1 ± 2.3	
IHD						
No (189)	30.9 ± 1.3	*p* = 0.64	29.9 ± 1.4	*p* = 0.77	30.4 ± 1.3	*p* = 0.67
Yes (12)	30.7 ± 1.5		29.8 ± 1.5		30.2 ± 1.4	
OSA						
No (196)	30.9 ± 1.4	*p* = 0.02	29.9 ± 1.5	*p* = 0.03	30.4 ± 0.8	*p* = 0.07
Yes (7)	31.1 ± 0.8		30.0 ± 0.8		31.1 ± 2.3	

OST differences between categoric variables of affected, unaffected, and healthy control right eyes (using t-test of independent variables). Abbreviations: HTN, hypertension; DM, diabetes mellitus; CVA, cerebral vascular accident; TIA, transient ischemic attack; IHD, ischemic heart disease; OSA, obstructive sleep apnea.

**Table A4:** Multivariate logistic regression analysis for OST and significant confounding variables.

	Dependent Variable: OST in the Medial Canthal Region			
Variable	OR	95% CI		*p*-Value
		Lower	Upper	
Room Temperature (°C)	2.1	1.56	2.72	<0.001
CVA/TIA (1 = Yes; 0 = No)	13.5	1.51	121	0.02
OST(°C)	0.93	0.68	1.26	0.63
	Dependent variable: OST in the Central Corneal region			
Variable	OR	95% CI		*p*-Value
		Lower	Upper	
Room Temperature (°C)	2.05	1.56	2.71	<0.001
CVA/TIA (1 = Yes; 0 = No)	13.8	1.54	122.9	0.02
OST(°C)	0.95	0.7	1.28	0.72
	Dependent variable: OST in the Lateral Canthal region			
Variable	OR	95% CI		*p*-Value
		Lower	Upper	
Room Temperature (°C)	2.2	1.67	2.92	<0.001
OST(°C)	0.76	0.53	1.0	0.08

Multivariate logistic regression analysis for OST and significant confounding variables. The comparison was made between the right eye from the control group and the RVO-affected and unaffected eyes. Multivariate logistic regression with adjustment for significant variables from full logistic regression: room temperature and also CVA for medial and central regions. Abbreviations: OST, ocular surface temperature; CI, confidence interval; OR, odds ratio. Significant confounders are bolded.

**Table A5:** Descriptive statistics of OST measurements (case–control): RVO-affected eyes vs. healthy controls.

Region	Medial	Central	Lateral
RVO	31.2 ± 1.65	30.4 ± 1.67	30.7 ± 1.65
Healthy controls	30.6 ± 1.32	29.5 ± 1.44	30.1 ± 1.48
*p*-value	*p* = 0.24	*p* = 0.1	*p* = 0.44

Descriptive statistics of OST measurements at all corneal regions ± SD and *p*-value of case–control comparison between RVO-affected eyes and healthy controls.

**Table A6:** Descriptive statistics of OST measurements (case–control): RVO-affected vs. unaffected eyes.

Region	Medial	Central	Lateral
Unaffected eyes	31.3 ± 1.72	30.2 ± 1.75	30.5 ± 1.71
Healthy controls	30.6 ± 1.32	29.5 ± 1.44	30.1 ± 1.48
*p*-value	*p* = 0.26	*p* = 0.25	*p* = 0.82

Descriptive statistics of OST measurements in all corneal regions ± SD and *p*-value of case–control comparison between RVO-unaffected eyes and healthy controls.
